# Sustainable green synthesis of CeO_2_–ZnO nanocomposites using *Rhazya stricta*: enhanced synergistic solutions for biomedical challenges and environmental remediation

**DOI:** 10.1039/d6ra02267g

**Published:** 2026-05-06

**Authors:** Amjid Khan, Tauqeer Ahmed Qadri, Rashid Abbas Khan, Dilawar Hassan, Bushra Ashiq, Ayesha Sani, Zabta Khan Shinwari, Malik Maaza

**Affiliations:** a UNESCO-UNISA Africa Chair in Nanosciences and Nanotechnologies, College of Graduate Studies, University of South Africa 1 Preller Street, Muckleneuk Ridge, P.O. Box 392 Pretoria Gauteng Province 0003 South Africa khana2@unisa.ac.za maazam@unisa.ac.za; b Nanosciences African Network (NANOAFNET), Materials Research Department, iThemba LABS-National Research Foundation, Western Cape Province South Africa; c African Centre of Competencies in Enhanced Nanosciences & Nanotechnologies for SDGs (ACCENTS) 1 Preller Street, Muckleuneuck Ridge, P.O. Box 392 Pretoria Gauteng Province 0003 South Africa; d Department of Biosciences, COMSATS University Islamabad Islamabad-45550 Pakistan; e Department of Biomedical Engineering, Research Center for Nano-biomaterials & AMP; Regenerative Medicine, College of Artificial Intelligence, Taiyuan University of Technology Taiyuan 030024 PR China; f Department of Plant Sciences, Faculty of Biological Sciences, Quaid-i-Azam University Islamabad 45320 Pakistan

## Abstract

Addressing the global demand for eco-friendly technologies, this study reports a sustainable ‘green’ route for the synthesis of cerium oxide (CeNPs), zinc oxide (ZnNPs), and their synergistic Ce–Zn nanocomposite (Ce–Zn Nc) using *Rhazya stricta* leaf extract. By replacing hazardous chemical reductants with renewable phytochemicals, this approach directly aligns with UN Sustainable Development Goal (SDG) 12 (Responsible Consumption and Production). GC-MS profiling identified 24 bioactive stabilizers, including Palmitic acid and Quebrachamine, which eliminate the need for toxic synthetic capping agents. Structural characterization (UV-Vis, XRD, FTIR, Raman, SEM/TEM) confirmed the successful synthesis of highly stable, crystalline particles, with the Ce–Zn Nc exhibiting a superior reduced average size of 10.7 nm and enhanced thermal stability compared to individual nanoparticles. Critically, the nanocomposite demonstrated enhanced synergistic efficiency in addressing challenges related to SDG 3 (Good Health) and SDG 6 (Clean Water). The Ce–Zn Nc showed superior antioxidant potential (Total Flavonoid Content: 342 ± 2.4 µg QE per mg) and robust antibacterial activity (26.7 ± 1.5 mm against *E. coli*). Furthermore, the composite provided an effective solution for environmental remediation, achieving 67.3 ± 4% catalytic degradation of methylene blue dye under solar irradiation. Hemolytic assays revealed a dose-dependent activity peaking at 59.1 ± 1.3%, indicating that while these materials possess significant membrane-disrupting potential, they offer a specialized bio-active alternative to traditional industrial catalysts. This research provides an evidence-based framework for scaling up multifunctional, bio-inspired nanomaterials to solve pressing sustainability challenges. Future research should focus on the multi-cycle stability and pilot-scale optimization of these green-synthesized nanocomposites to facilitate their practical implementation in industrial environmental remediation and clinical diagnostics.

## Abstract 2

Sustainable green synthesis of CeNPs, ZnNPs, and Ce–Zn nanocomposites using *Rhazya stricta* for enhanced synergistic solutions in solar-driven dye degradation (SDG 6) and antimicrobial/biomedical applications (SDG 3).

## Graphical abstract

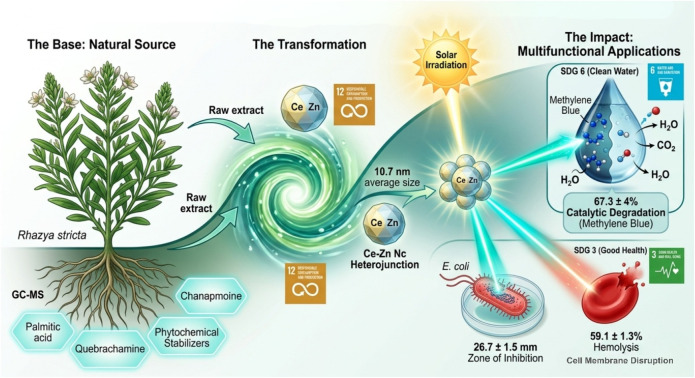

## 1. Introduction

Nanotechnology has transformed various scientific domains by enabling the synthesis of materials with distinct properties at the nanoscale.^1–3^ This interdisciplinary field has driven progress in medicine, environmental science, energy, and electronics by developing materials with chemical reactivity, electrical conductivity, optical features, and biological activity. In particular, nanoparticles like cerium oxide (CeO_2_) and zinc oxide (ZnO) have attracted attention because of their remarkable catalytic, antioxidant, antimicrobial, and optical attributes, making them highly valuable in biomedical fields, environmental cleanup, and industrial applications.^4–6^ Additionally, nanotechnology aligns with key United Nations Sustainable Development Goals (SDGs), such as SDG 3 (Good Health and Well-being), SDG 6 (Clean Water and Sanitation), SDG 7 (Affordable and Clean Energy), and SDG 12 (Responsible Consumption and Production).^7–12^ For example, nanomaterials improve drug delivery systems, enhance healthcare outcomes, optimize water purification methods, and contribute to sustainable energy solutions. However, conventional synthesis methods of nanoparticles, typically involving physical and chemical processes, are often energy-intensive and rely on toxic chemicals that pose environmental and health hazards, thereby conflicting with the principles of sustainable development and limiting large-scale, eco-friendly production.^13^

To overcome these limitations, green synthesis methods utilizing biological resources like plants, bacteria, fungi, and algae have emerged as environmentally friendly alternatives.^14–16^ Among these methods, plant-mediated synthesis is particularly appealing due to the variety of bioactive phytochemicals it involves, such as phenols, flavonoids, alkaloids, and terpenoids, which act as natural agents for reducing and stabilizing nanoparticles.^17–19^ Recent high-impact literature has significantly expanded the scope of these green-synthesized materials, demonstrating their versatility in complex electrochemical and environmental systems. For instance, the fabrication of carbon-based 4d bimetallic ZnO nanocomposites has shown exceptional sensitivity in the detection of pharmaceutical compounds like phenylephrine hydrochloride, highlighting the role of bimetallic interfaces in enhancing electron transfer.^20^ Similarly, the use of sago starch as a capping agent for CuO nanorods impregnated in soy protein matrices has pioneered the development of biodegradable nano-biocomposites for the detection of cytosine.^21^ Furthermore, the integration of silver-iron oxide (Ag–Fe_3_O_4_) nanoparticles into collagen-based matrices derived from biological waste underscores the shift toward multifunctional, magnetized biomaterials capable of both antibacterial action and water purification.^22^ Furthermore, the application of green-synthesized bimetallic nanoparticles has recently been shown to play a pivotal role in climate-resilient agriculture by mitigating heavy metal toxicity, such as nickel (Ni) stress, through the modulation of antioxidant defense systems and the restriction of metal translocation in staple crops like wheat.^23^ These studies emphasize that the synergy between a stabilizer (matrix) and a metallic heterojunction is key to overcoming the limitations of monometallic systems. This approach reduces the need for harmful chemicals and energy-intensive processes, often resulting in nanoparticles with improved biocompatibility and enhanced biological functionalities.^24,25^ However, green synthesis also has challenges, including variability in phytochemical composition, batch-to-batch inconsistency, and sometimes less control over nanoparticle size and shape compared to chemical methods.^26–28^

Despite these advancements, the specific role of *Rhazya stricta* metabolites in directing the growth of CeO_2_–ZnO heterostructures remains unexplored. While chemical synthesis offers precise control, it frequently compromises biocompatibility.^27^*Rhazya stricta*, a medicinal plant rich in alkaloids, presents a promising bioresource.^28^ The novelty of the present study lies in its GC-MS-guided approach, which moves beyond empirical “trial-and-error” synthesis to specifically correlate identified metabolites, such as quebrachamine and palmitic acid, with the structural evolution of the Ce–Zn nanocomposite. By integrating detailed phytochemical profiling with the systematic evaluation of synergistic antioxidant, antimicrobial, and catalytic efficiencies, this research provides a mechanistic framework that addresses the reproducibility and performance gaps highlighted in recent literature.^27,29^ This approach is coupled with an extensive evaluation of multifunctional properties, including antioxidant, antimicrobial, catalytic, and hemolytic biocompatibility, highlighting the material's potential in bridging the gap between sustainable phytochemistry and high-performance nanotechnology for biomedical and environmental remediation.

The novelty of this research lies in its Gas Chromatography-Mass Spectrometry (GC-MS) guided approach, which correlates identified metabolites from *Rhazya stricta*, such as quebrachamine and palmitic acid, with the structural evolution and synergistic stabilization of cerium oxide–zinc oxide (CeO_2_–ZnO) nanocomposites. Moving beyond empirical synthesis, this work aims to characterize the resulting 10.73 nm particles using advanced techniques, including Ultraviolet-Visible (UV-Vis) spectroscopy, X-ray diffraction (XRD), Fourier Transform Infrared (FTIR) spectroscopy, Raman scattering, Thermogravimetric Analysis-Differential Scanning Calorimetry (TGA-DSC), Scanning Electron Microscopy (SEM), and Transmission Electron Microscopy (TEM), to establish a mechanistic link between phytochemistry and material performance. By systematically evaluating antioxidant, antimicrobial, and catalytic activities alongside hemolytic biocompatibility, this research addresses the reproducibility gaps in sustainable nanotechnology. Ultimately, these findings highlight the potential of bio-inspired heterostructures as multifunctional tools for high-performance biomedical and environmental applications.^30^

## 2. Materials and methods

### 2.1. Plant collection and extract preparations

**Fig. 1:**
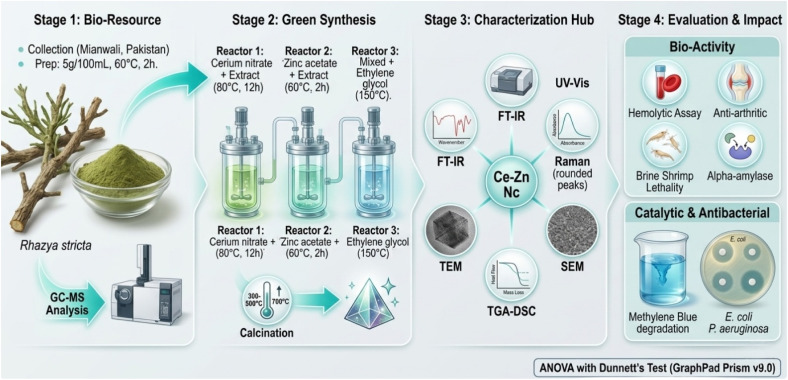
Schematic illustration of the experimental methodology for the phytochemical profiling and eco-friendly synthesis of cerium oxide (CeNPs), zinc oxide (ZnNPs), and cerium–zinc nanocomposites (Ce–Zn Nc) using *Rhazya stricta*.

The study was conducted at University of South Africa, South Africa. The *Rhazya stricta* leaves were collected in June 2022 from Village Sawans, District Mianwali, Punjab Province, Pakistan, at coordinates 32°43′44″ N, 71°37′59″ E, with an elevation of 285 meters above sea level. This collection was performed on private land, with explicit permission from the landowner. All activities adhered to institutional, national, and international ethical guidelines for the collection of plant materials. The taxonomic identification was carried out at QAU by Prof. Dr Muhammad Zafar, an expert taxonomist. A voucher specimen (No. 133569) was deposited in the Herbarium of Pakistan (ISL) for future reference. The plant material was thoroughly washed with deionized water, dried, and ground. For extract preparation, 5 g of dried powder was added to 100 mL of distilled water and stirred at 60 °C for 2 h. The resulting extract was filtered through Whatman No. 1 paper and stored at 4 °C for nanoparticle synthesis and other applications. Fig. 1 provides a schematic illustration of the experimental methodology for the phytochemical profiling and eco-friendly synthesis of cerium oxide (CeNPs), zinc oxide (ZnNPs), and cerium-zinc nanocomposites (Ce–Zn Nc) using *Rhazya stricta*. It outlines the key processes involved in the synthesis, characterization, and bioactivity testing.

### 2.2. Phytochemical characterization

Ethanol extracts of *R*. *stricta* leaves were analyzed using gas chromatography-mass spectrometry (GC-MS) on an Agilent 7890A GC system coupled with an MSD-5975C mass selective detector. Separation was carried out using an HP-5MS column. The compounds were identified by comparing their mass spectra with standard databases, including the Wiley Registry/NIST Mass Spectral Library (2023) and Adams libraries.^31^

### 2.3. Biosynthesis of cerium oxide nanoparticles (CeNPs)

CeNPs were synthesized following a modified method from.^32^ Briefly, 7.43 g of cerium nitrate hexahydrate (Ce(NO_3_)_3_·6H_2_O) was dissolved in 100 mL of distilled water and stirred for 40 min. Then, 20 mL of *R. stricta* leaves extract was slowly added to the cerium nitrate solution while maintaining a temperature of 80 °C for 6 h. The resulting lemon-colored gel was dried at 60 °C for 6 h and subsequently heated at 300, 400, and 500 °C for 2 h to produce cerium oxide nano-powder.

### 2.4. Biosynthesis of zinc oxide nanoparticles (ZnNPs)

To synthesize ZnNPs, 6.0 g of zinc acetate dihydrate was added to 100 mL of *R. stricta* leaves extract and heated with constant stirring at 60 °C for 2 h. The pH of the mixture was monitored to ensure proper nanoparticle formation. The mixture was then centrifuged at 10 000 rpm for 20 min at 40 °C. The resulting pellets were washed twice with deionized water, dried in an oven at 100 °C, and crushed into a fine powder. The powder was then annealed at temperatures ranging from 100 to 500 °C for 2 h to enhance crystallinity, following the method modified from.^33^

### 2.5. Synthesis of cerium and zinc oxide nanocomposites (Ce–Zn Nc)

Ce–Zn Nc were synthesized using a green approach adapted from.^34^ A stoichiometric amount of zinc nitrate hexahydrate (Zn(NO_3_)_2_·6H_2_O) was dissolved in 150 mL of deionized water. Separately, 30 mL of cerium nitrate solution was prepared and added dropwise to the zinc solution under constant stirring for one hour. Then, 10 mL of ethylene glycol and 10 mL of *R. stricta* leaves were extracted added sequentially, and the mixture was stirred continuously. The color change to brown indicated successful nanocomposite formation. The mixture was filtered, dried at 150 °C, and calcinated at 700 °C for 4 h in a muffle furnace to obtain Ce–Zn Nc powders.

### 2.6. Characterizations of CeNPs, ZnNPs, and Ce–Zn Nc

UV-Vis spectroscopy confirmed nanoparticle formation. Post-centrifugation and drying, nanoparticles were characterized by Fourier-transform infrared spectroscopy (FTIR), and Raman spectroscopy for structural and functional group analysis. Surface plasmon resonance peaks were recorded *via* UV-Vis (UV-2600) and characterization involved X-ray diffraction (XRD) (Model: D8 Advance, Bruker, Germany) to evaluate structural properties. High-resolution SEM and TEM examined morphological features, and thermal stability was analyzed by thermogravimetric analysis coupled with differential scanning calorimetry (TGA-DSC).

### 2.7. Phytochemical screening and antioxidant assay

The total phenolic content (TPC) of *R. stricta* extract, CeNPs, ZnNPs, and Ce–Zn Nc (25, 50, and 100 µg mL^−1^) was measured using the Folin and Ciocalteu methods.^35^ The total flavonoid content (TFC) was determined using the aluminum chloride colorimetric method,^35^ and total antioxidant capacity (TAC) was measured using phosphomolybdenum method.^35^ The ferric reducing antioxidant power (FRAP) was evaluated using a modified version of a previously established method.^35^

### 2.8. Antioxidant free radical scavenging assay

The antioxidant activity of *R. stricta* extract, CeNPs, ZnNPs, and Ce–Zn Nc was tested using DPPH (2,2-diphenyl-1-picrylhydrazyl), and ABTS^•+^ [2,2′-azino-bis (3-ethylbenzothiazoline-6-sulfonic acid)] free radical scavenging method.^36^ The radical scavenging percentage (RSA%) was calculated as:• RSA (%) = [ (Abs_control_ − Abs_sample_)/Abs_control_] × 100

H_2_O_2_ scavenging activity was determined similarly.

### 2.9. Bioactivity and toxicity evaluation assays

The bioactivity and toxicity of *R. stricta* extract, CeNPs, ZnNPs, and Ce–Zn Nc were evaluated through various assays. The hemolytic activity was assessed using RBCs, with Triton X-100 as the positive control and DMSO as the negative control.^37^ The anti-arthritic activity was measured by turbidity, based on the method by.^38^ Brine shrimp lethality was determined using a modified method^39^ with doxorubicin as the positive control. Alpha-amylase inhibition was evaluated using acarbose as the control.^40^ The following formulas were used to calculate the results for each assay:• Hemolysis (%) = [(Abs_control_ − Abs_sample_)/Abs_control_] × 100• Inhibition (%) = [*A*_sample_/*A*_control_] × 100• Lethality (%) = [ (Nauplli death_before treatment_ − Nauplli death_after treatment_)/Nauplli death_before treatment_] × 100

### 2.10. Catalytic activity

The catalytic efficiency of *R. stricta* extract, CeNPs, ZnNPs, and Ce–Zn Nc was evaluated by methylene blue (MB) degradation, using a modified method from by.^41^ The catalytic degradation efficiency was calculated using the following formula:• Degradation (%) = 100 × (*A*_0_ − *A*)/*A*_0_

### 2.11. Antibacterial activity

A modified disc diffusion method^42^ was used to assess the antibacterial activity of *R. stricta* extract, CeNPs, ZnNPs, and Ce–Zn nanocomposites against *Pseudomonas aeruginosa*, *Escherichia coli*, and *Klebsiella pneumoniae*.^43^ Bacterial cultures (1 × 10^8^ CFU mL^−1^) were spread on TSA plates, and 5 mm discs loaded with nanoparticle suspensions (25–100 µg mL^−1^) were applied. Ampicillin served as a positive control. Plates were incubated at 37 °C for 24 h, and zones of inhibition were measured.

### 2.12. Statistical analysis

All experiments were performed in triplicate (*n* = 3), and results were expressed as mean ± standard deviation. Statistical analysis was carried out using GraphPad Prism version 9.0.0, applying two-way ANOVA followed by Dunnett's multiple comparisons test. A *p*-value < 0.05 was considered statistically significant.

## 3. Results

### 3.1. GC-MS profiling of ethanolic leaf extract of *R*. *stricta*

The GC-MS analysis of the 96% ethanolic leaf extract of *R*. *stricta* identified 25 phytochemical compounds with diverse pharmacological activities (Table 1). These compounds exhibited retention times ranging from 12.42 to 27.06 minutes and possessed various molecular formulas indicative of their chemical nature. Significant bioactive compounds included propanoic acid, 2-methyl-, 2,2-dimethyl-1-(2-hydroxy-1-methylethyl) propyl ester (C_14_H_28_O_3_) with a retention time of 12.42 minutes, which is known for potent antimicrobial and antifungal effects, making it valuable in personal care formulations. Antioxidant compounds such as 2-(2-hydroxyphenoxy)-1-phenyl ethanol (C_14_H_14_O_3_, RT 13.84 min) and 2,4-di-*tert*-butylphenol (C_17_H_30_OSi, RT 14.45 min) were also detected and commonly used in pharmaceutical products to prevent oxidative degradation. Fatty acids and their esters, like *n*-hexadecanoic acid (C_16_H_32_O_2_, RT 15.03 min), palmitic acid ethyl ester (C_18_H_36_O_2_, RT 19.55 min), linolenic acid (C_18_H_30_O_2_, RT 21.02 min), and stearic acid (C_18_H_36_O_2_, RT 21.17 min) were present, which contribute anti-inflammatory properties and are frequently used in lipid-based drug delivery systems. Cyclic hydrocarbons such as cyclododecane (C_12_H_24_, RT 16.30 min) and cyclotridecane (C_13_H_26_, RT 17.42 min), along with terpenoids like pinane, cis (C_10_H_18_, RT 18.01 min) and phytol (C_20_H_40_O, RT 20.71 min), were also identified, indicating roles in stabilization and anti-inflammatory effects. Alkaloids with potential therapeutic effects, including (−)-eburenine (C_19_H_24_N_2_, RT 22.11 min), aspidospermidine (C_19_H_26_N_2_, RT 22.48 min), and quebrachamine (C_19_H_26_N_2_, RT 24.37 min), were detected, suggesting possible anticancer, neuroprotective, and analgesic activities. This diverse phytochemical profile supports the medicinal value of *R. stricta* leaves and provides a chemical foundation for their biological activities observed in further studies.

**Table 1:** Phytochemical constituents of the *R. stricta* 96% ethanolic leaf extract

S. No	Name of the compound	Molecular formula	Molecular weight	RT (min)	Pharmacological activities
1	Propanoic acid, 2-methyl-, 2,2-dimethyl-1-(2-hydroxy-1-methylethyl) propyl ester	C_14_H_28_O_3_	216.173	12.42	This compound exhibits potent antimicrobial and antifungal activities, making it valuable in personal care formulations. It also serves as a potential agent for skin-related disorders due to its broad-spectrum effectiveness
2	2-(2-Hydroxyphenoxy)-1-phenyl ethanol	C_14_H_14_O_3_	230.094	13.841	Used as an antioxidant in pharmaceutical formulations and in skincare products for its moisturizing and anti-aging effects
3	2,4-Di-*tert*-butylphenol	C_17_H_30_OSi	206.167	14.451	Used as an antioxidant in pharmaceutical formulations to prevent oxidative degradation of sensitive drugs
4	*n*-Hexadecanoic acid	C_16_H_32_O_2_	256.24	15.03	Used in lipid-based drug delivery systems and as an anti-inflammatory agent in the treatment of chronic conditions like cardiovascular diseases
5	2,3,5,6-Tetrafluoroanisole	C_7_H_4_F_4_O	180.02	15.188	Used in the synthesis of fluorinated drugs to enhance bioactivity, stability, and receptor binding
6	Cyclododecane	C_12_H_24_	168.188	16.303	Used in drug delivery systems and biological sample preservation due to its ability to stabilize hydrophobic compounds and provide controlled release
7	Cyclotridecane	C_13_H_26_	182.203	17.418	Used in controlled drug delivery systems and for stabilizing sensitive biological compounds, aiding in long-term preservation
8	Pinane, cis	C_10_H_18_	138.141	18.007	Used in pharmaceutical formulations for its anti-inflammatory and analgesic properties, particularly in topical treatments for pain relief
9	4-Phenylpyrazole	C_9_H_8_N_2_	144.069	18.207	Used in the development of insecticides and as a model compound in pharmaceutical research for parasitic diseases
10	Femerazol	C_10_H_10_N_2_	158.084	18.776	Used in research for its potential anti-inflammatory and antimicrobial properties, particularly in treating parasitic infections
11	Palmitic acid ethyl ester	C_18_H_36_O_2_	284.272	19.554	Used in lipid-based drug delivery systems to improve the solubility and bioavailability of lipophilic drugs
12	1,9-Tetradecadiene	C_14_H_26_	194.203	20.291	Used in the synthesis of bioactive compounds and as a precursor in the development of targeted drug delivery systems
13	7-Oxabicyclo [4.1.0] heptane, 3-oxiranyl-	C_9_H_16_O_2_	140.084	20.606	Used in drug development as a structural scaffold for designing bioactive molecules with enhanced stability and targeted therapeutic effects
14	Phytol	C_20_H_40_O	296.308	20.712	Used in the synthesis of vitamin E and other bioactive compounds, and in the development of anti-inflammatory and antioxidant formulations
15	Linolenic acid	C_18_H_30_O_2_	278.225	21.017	Used in the development of anti-inflammatory, cardiovascular, and neuroprotective treatments due to its omega-3 fatty acid properties
16	Stearic acid	C_18_H_36_O_2_	284.272	21.175	Used in pharmaceutical formulations as a stabilizer, emulsifier, and in lipid-based drug delivery systems for controlled release
17	(−)-Eburenine	C_19_H_24_N_2_	280.41	22.111	Investigated for its potential anticancer and neuroprotective properties, particularly in the development of novel therapeutic agents
18	Aspidospermidine	C_19_H_26_N_2_	282.21	22.479	Investigated for its anticancer, anti-inflammatory, and analgesic properties, and used in the development of natural drug compounds
19	1-[2-Pyridyl]-2,2-dimethyl-2-piperidino ethanol	C_7_H_15_NO	234.173	23.489	Explored in neuropharmacology for its potential role in targeting specific receptors in the central nervous system for therapeutic effects
20	2-Methylresorcinol, diacetate	C_11_H_12_O_4_	208.074	23.847	Used in skincare products for its antioxidant, anti-inflammatory, and skin-brightening properties, especially in treating hyperpigmentation
21	Quebrachamine	C_19_H_26_N_2_	282.21	24.373	Investigated for its potential anticancer properties, particularly in inhibiting cancer cell growth and developing novel cancer therapies
22	Diazepam-d5	C_16_H_13_ClN_2_O	265.122	24.805	Used in pharmacokinetic studies to trace the metabolism and distribution of diazepam, aiding in the development of benzodiazepine-based therapies
23	Succinic acid, heptyl 4-methoxyphenyl ester	^C^_18_H_34_O_4_	322.178	25.373	Used in drug formulations as an intermediate for synthesizing bioactive compounds with potential anti-inflammatory and analgesic properties
24	2,6,10,14,18,22-Tetracosahexaene, 2,6,10,15,19,23-hexamethyl-, (all-E)-	C_30_H_50_	410.391	26.614	Used in research related to polyunsaturated fatty acids, focusing on their role in cellular signaling, oxidative stress management, and inflammation-related diseases
25	Acetic acid, 6-morpholin-4-yl-9-oxobicyclo [3.3.1] non-3-yl ester	C_12_H_22_O_2_	281.163	27.056	Investigated for its potential anticancer properties, acting as an enzyme inhibitor to target cancer growth and proliferation

### 3.2. Characterization of nanoparticles

The green synthesis of CeNPs, ZnNPs, and Ce–Zn Nc was confirmed and characterized using a series of analytical techniques, including UV-visible spectroscopy, Fourier-transform infrared spectroscopy (FTIR), Raman spectroscopy, X-ray diffraction (XRD), thermogravimetric analysis–differential scanning calorimetry (TGA-DSC), scanning electron microscopy (SEM), and high-resolution transmission electron microscopy (HR-TEM).

### 3.3. UV-Vis, FTIR, and Raman spectroscopy

**Fig. 2:**
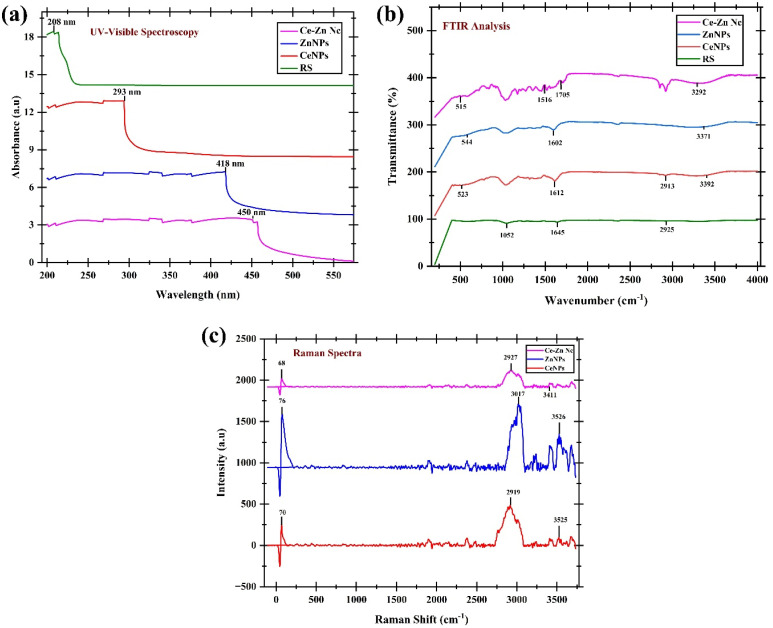
(a) UV-Visible absorption spectra, (b) Fourier Transform Infrared (FTIR) spectra, and (c) Raman spectra of *Rhazya stricta* extract (RS), cerium oxide nanoparticles (CeNPs), zinc oxide nanoparticles (ZnNPs), and cerium–zinc nanocomposites (Ce–Zn Nc).

The UV-Visible absorption spectra of *R. stricta* extract (RS), CeNPs, ZnNPs, and their Ce–Zn Nc were recorded between 200 and 550 nm to confirm nanoparticle synthesis and assess their optical properties, as shown in Fig. 2a. The RS extract exhibited a prominent absorption peak at 208 nm, attributable to its phytochemical constituents responsible for the reduction and stabilization of metal ions during nanoparticle formation. CeNPs displayed a characteristic surface plasmon resonance (SPR) peak at 293 nm, confirming the successful biosynthesis of cerium oxide nanoparticles. ZnNPs showed an intense absorption peak at 418 nm, corresponding to the typical SPR of zinc oxide nanoparticles. The Ce–Zn nanocomposites demonstrated a red-shifted SPR peak at 450 nm compared to ZnNPs, indicating the formation of a bimetallic nanocomposite with modified electronic interactions between cerium and zinc oxide species. These distinct absorption features validate the effective green synthesis of the nanoparticles using *R. stricta* extract and highlight the extract's role as both reducing and capping agent, leading to the formation of stable nanostructures with unique optical properties.

The FTIR spectra of RS, CeNPs, ZnNPs, and CeZn-Nc were analyzed to identify the functional groups responsible for the reduction and capping of the nanomaterials (Fig. 2b and Table S1). The RS extract displayed prominent peaks at 3292 cm^−1^ (O–H stretching) and 2925 cm^−1^ (C–H stretching), corresponding to the polyphenols and alkanes present in the phytochemical profile. The band at 1645 cm^−1^ represents the C=O/N–H stretching of amides, while the peak at 1052 cm^−1^ is attributed to C–O stretching vibrations of alcohols and ethers. Following the synthesis of nanoparticles, the characteristic hydroxyl band shifted to 3392 cm^−1^ for CeNPs and 3371 cm^−1^ for ZnNPs, indicating the active participation of phenolic groups in metal ion reduction. The formation of metal–oxygen bonds was confirmed by the appearance of sharp peaks in the fingerprint region: the band at 544 cm^−1^ corresponds to the Zn–O stretching vibration, while the peak at 523 cm^−1^ represents Ce–O stretching. In the CeZn-Nc spectrum, the formation of the heterojunction is evidenced by the shift in metal–oxygen vibrations to 515 cm^−1^ (M–O stretching). Additionally, the presence of aromatic C=C stretching at 1516 cm^−1^ and carboxylic C=O stretching at 1705 cm^−1^ in the nanocomposite confirms that the *R. stricta* metabolites effectively stabilized the bimetallic system.

The structural properties and vibrational modes of the synthesized nanomaterials were investigated using Raman spectroscopy (Fig. 2c and Table S2). In the low-frequency region, distinct sharp peaks were observed at 70 cm^−1^ (CeNPs), 76 cm^−1^ (ZnNPs), and 68 cm^−1^ (CeZn-Nc), which are characteristic of the lattice vibrations (phonons) of the metal oxide frameworks. The presence of phytochemical capping agents from the RS extract is further evidenced by the C–H stretching vibrations observed in the 2919–3017 cm^−1^ range and O–H/N–H stretching modes at 3411–3526 cm^−1^. By removing the environmental CO_2_ background signals at ∼2380 cm^−1^ as suggested, the Raman profile confirms a high degree of crystallinity and successful stabilization of the CeZn-Nc heterojunction by the plant secondary metabolites.

### 3.4. XRD pattern

**Fig. 3:**
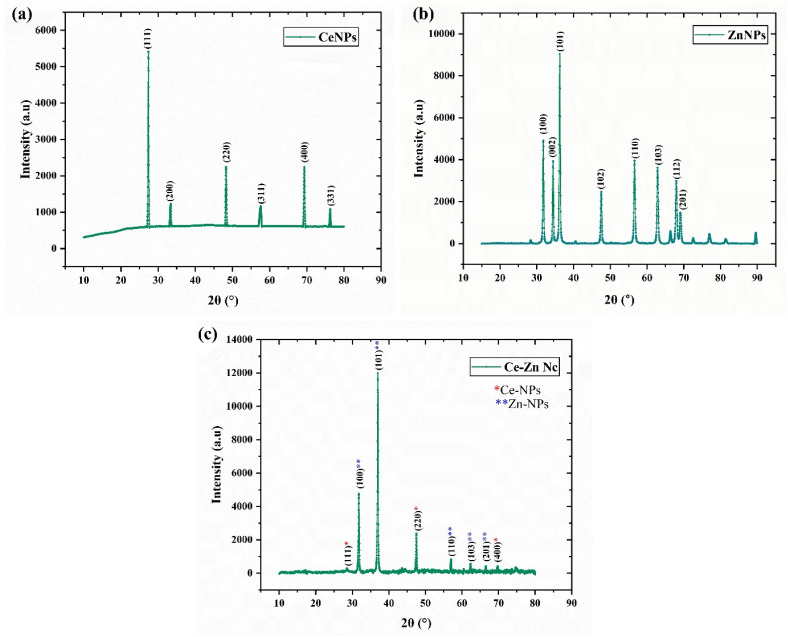
X-ray diffraction (XRD) patterns of green-synthesized nanomaterials. (a) Pure CeNPs exhibiting cubic fluorite structure, (b) pure ZnNPs exhibiting hexagonal wurtzite structure, and (c) Ce–Zn Nc demonstrating the successful formation of a bimetallic heterojunction. Peak indexing corresponds to JCPDS Card Nos. 00-034-0394 (CeO_2_), 00-036-1451 (ZnO), and 00-043-1002 (composite phase). Asterisks in (c) denote the overlapping characteristic reflections from both constituent phases, confirming high phase purity and crystallinity.

The crystalline phases and structural integrity of the green-synthesized nanomaterials were rigorously analyzed *via* X-ray diffraction, with all patterns indexed against standard JCPDS references to confirm phase purity (Fig. 3). For the CeNPs (Fig. 3a), the diffraction peaks observed at 2*θ* values of 28.5°, 33.1°, 47.5°, 56.3°, 69.4°, and 76.7° correspond to the (111), (200), (220), (311), (400), and (331) crystallographic planes, respectively, confirming a pure cubic fluorite structure (JCPDS Card No. 00-34-0394). The ZnNPs pattern (Fig. 3b) exhibited sharp reflections at 2*θ* = 31.8°, 36.3°, 47.5°, 56.6°, 62.8°, 67.9°, and 69°, which align with the (100), (002), (101), (102), (110), (103), (112) and 201 planes of the hexagonal wurtzite ZnO phase (JCPDS Card No. 00-036-1451). Critically, the pattern for the Ce–Zn Nc (Fig. 3c) validates the successful formation of a bimetallic heterojunction, as it simultaneously displays the characteristic diffraction signatures of both CeO_2_ and ZnO frameworks, consistent with the composite phase indexing of JCPDS Card No. 00-043-1002. The absence of extraneous peaks indicates that the phytochemicals in the *R*. *stricta* extract acted as effective stabilizing agents, preventing the formation of impurity phases or precursor residues. Furthermore, the notable peak broadening in the nanocomposite suggests a reduction in crystallite size compared to the individual nanoparticles, facilitating a high surface-area-to-volume ratio that significantly enhances the available active sites for the observed catalytic and antibacterial applications.

### 3.5. Analysis of nanoparticles *via* TGA-DSC

**Fig. 4:**
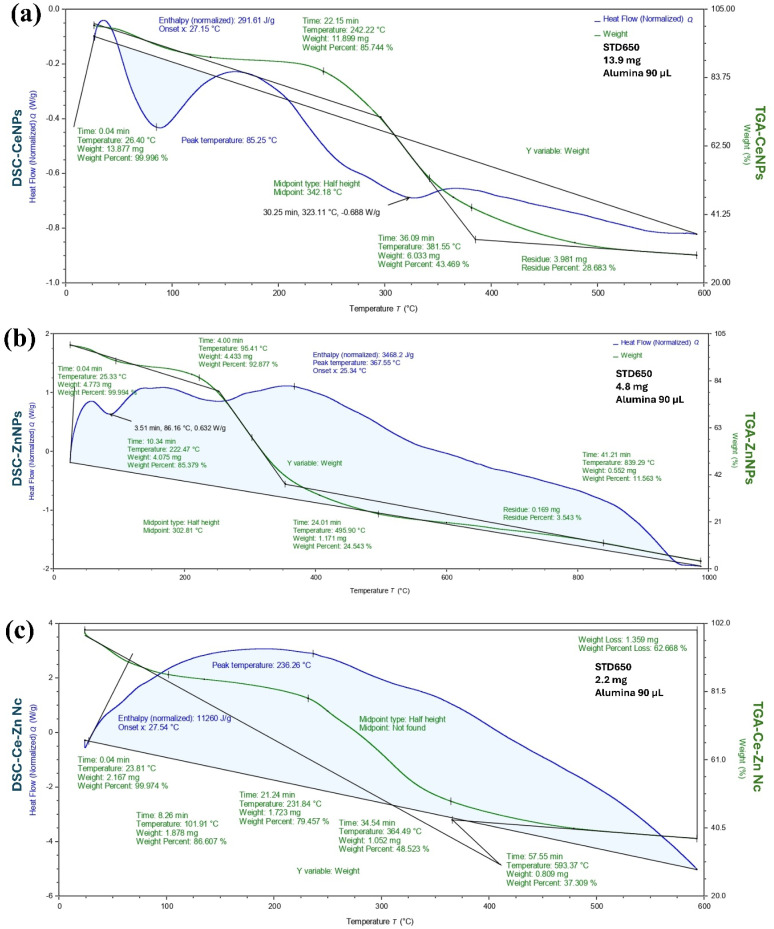
Thermogravimetric analysis (TGA) and differential scanning calorimetry (DSC) curves of (a) CeNPs, (b) ZnNPs, and (c) Ce–Zn Nc showing thermal stability and decomposition profiles.

The thermal stability and decomposition behavior of CeNPs, ZnNPs, and Ce–Zn Nc were evaluated using TGA and DSC analyses, as presented in Fig. 4. For CeNPs (Fig. 4a), the TGA curve showed an initial weight loss of approximately 14.3% occurring up to 242 °C, which corresponds to the evaporation of physically adsorbed water and volatile organic compounds. The DSC curve revealed an endothermic peak at 85.3 °C, attributed to moisture loss. A significant weight reduction (∼42.3%) was observed between 242 °C and 381.6 °C, likely due to the decomposition of organic capping agents and phytochemicals associated with the nanoparticles. The residual weight at 600 °C was about 28.7%, indicating good thermal stability of the CeNPs. In the case of ZnNPs (Fig. 4b), an initial weight loss of around 7.1% up to 95.4 °C was noted, associated with moisture evaporation. DSC analysis showed an endothermic peak at 86.2 °C. Further weight loss (∼61.9%) occurred between 95 °C and 839 °C, attributed to the degradation of organic components and possible structural transformations. The residual mass at 1000 °C was approximately 3.5%, indicating the presence of thermally stable zinc oxide. The Ce–Zn nanocomposite (Fig. 4c) exhibited a more complex thermal behavior, with an initial weight loss of about 13.4% up to 101.9 °C, related to moisture and volatile substances. The DSC curve displayed a notable peak at 236.3 °C, corresponding to the decomposition of organic moieties. Subsequent weight losses were observed at 231.8 °C and 364.5 °C, totaling approximately 62.7% by 600 °C. The residual mass of about 37.3% confirmed enhanced thermal stability due to the formation of the nanocomposite structure. Overall, the TGA-DSC results demonstrate that all synthesized nanomaterials possess good thermal stability, with the Ce–Zn nanocomposite showing improved resistance to thermal degradation, likely due to synergistic interactions between cerium and zinc oxides.

### 3.6. Morphological analysis of nanoparticles *via* SEM

**Fig. 5:**
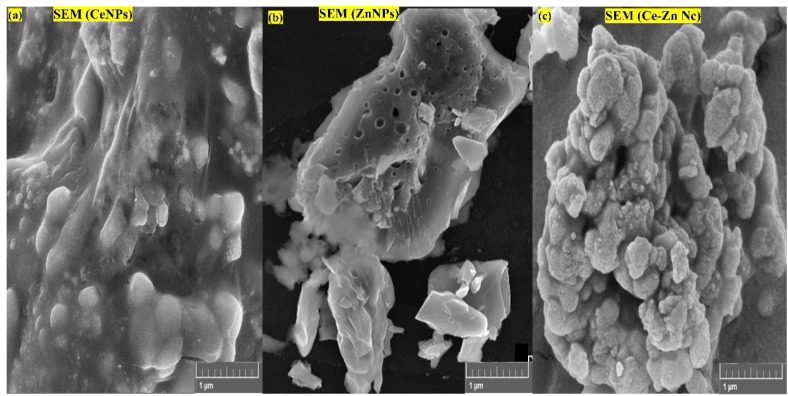
Scanning electron microscopy (SEM) images showing the surface morphology of (a) CeNPs, (b) ZnNPs, and (c) Ce–Zn Nc at 1 µm scale bar.

The surface morphology of the synthesized CeNPs, ZnNPs, and Ce–Zn Nc was examined using SEM, as shown in Fig. 5. The CeNPs exhibited an irregular, aggregated morphology with smooth surfaces and some degree of particle agglomeration, characteristic of nanocluster formation (Fig. 5a). The ZnNPs displayed a distinctly porous and flaky structure with well-defined edges, indicative of their high surface area and crystalline nature (Fig. 5b). In contrast, the Se–Zn nanocomposites formed dense, roughly spherical aggregates with a highly clustered and compact arrangement, suggesting strong interactions between cerium and zinc oxide phases in the composite (Fig. 5c). Collective morphology supports the successful synthesis and integration of metal oxides, with potential implications for enhanced surface reactivity and stability in multifunctional applications.

### 3.7. High-resolution TEM analysis of nanoparticles

**Fig. 6:**
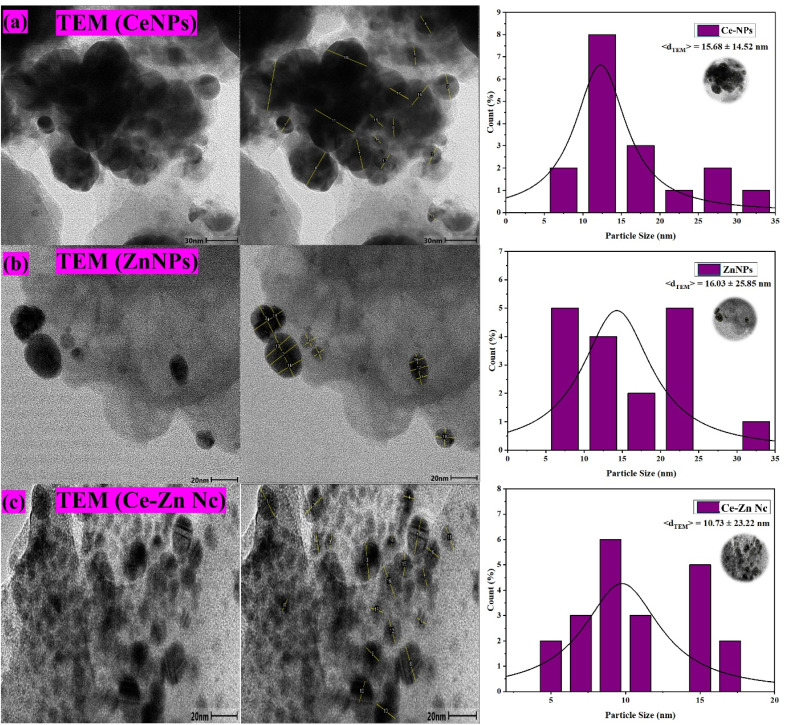
TEM images and particle size distribution histograms of (a) CeNPs, (b) ZnNPs, and (c) Ce–Zn Nc, along with TEM analysis showing lattice fringes confirming crystallinity and uniform composition.

TEM images of CeNPs, ZnNPs, and Ce–Zn Nc revealed well-dispersed nanoscale particles with varying morphologies, as shown in Fig. 6. The CeNPs appeared predominantly spherical with some agglomeration, and the particle size distribution histogram indicated an average diameter of 15.7 ± 14.5 nm (Fig. 6a). ZnNPs exhibited irregular shapes with slight clustering, and their particle size distribution showed an average size of 16 ± 25.9 nm (Fig. 6b), indicating a wider size distribution compared to CeNPs. The Ce–Zn nanocomposites displayed smaller, more uniformly distributed particles with an average diameter of 10.7 ± 23.2 nm (Fig. 6c), suggesting enhanced homogeneity in the bimetallic composite. TEM images revealed clear lattice fringes, confirming the crystalline nature of the nanoparticles. The compact, homogeneous morphology of Ce–Zn Nc suggests enhanced physicochemical properties. TEM analyses confirm the successful synthesis of nanoscale particles with controlled size and morphology, crucial for multifunctional applications.

### 3.8. Phytochemical content and antioxidant activity

#### 3.8.1. TFC, TFC, FRAP, and TAC

**Fig. 7:**
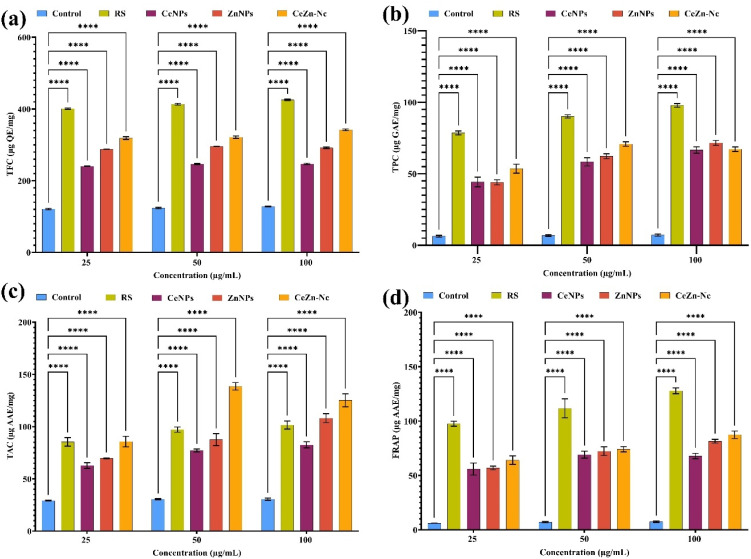
Comparative analysis of antioxidant and phytochemical contents of RS, CeNPs, ZnNPs, and CeZn-Nc at various concentrations (25, 50, 100 µg mL^−1^): (a) total flavonoid content (TFC, µg QE per mg), (b) total phenolic content (TPC, µg GAE per mg), (c) total antioxidant capacity (TAC, µg AAE per mg), and (d) ferric reducing antioxidant power (FRAP, µg AAE/mg). Data represent mean ± SD (*n* = 3). Statistical significance was determined by two-way ANOVA with Dunnett's multiple comparisons test (****p < 0.0001 *vs.* control).

Total flavonoid content (TFC) increased significantly in all treatment groups compared to the control at 25, 50, and 100 µg mL^−1^ concentrations (****p < 0.0001; Fig. 7a). At 100 µg mL^−1^, RS exhibited the highest TFC of 425.7 ± 2.0 µg QE per mg, representing a 167% increase over the control (128.1 ± 1.0 µg QE/mg). The Ce–Zn Nc showed a comparable enhancement (342 ± 2.4 µg QE per mg), while ZnNPs and CeNPs recorded increases of 127% and 93%, respectively. TFC in RS and Ce–Zn Nc was significantly higher than in the individual nanoparticles (*p* < 0.05). Similarly, total phenolic content (TPC) was significantly elevated across all treatments at all tested concentrations (*p* < 0.0001; Fig. 7b). RS demonstrated the highest TPC values, ranging from 78.6 ± 1.3 to 97.9 ± 1.3 µg GAE per mg, reflecting substantial increases of 1137% to 1269% over the control (6.4 ± 0.7 µg GAE per mg*). Ce–Zn Nc also exhibited significant enhancement (53.6 ± 3.1 to 67.3 ± 1.6 µg GAE per mg), whereas ZnNPs and CeNPs showed moderate but statistically significant increases.

Total antioxidant capacity (TAC) and ferric reducing antioxidant power (FRAP) similarly showed significant increases relative to control at all concentrations (****p < 0.0001, Fig. 7c and 6d). RS demonstrated the highest TAC (85.5 ± 4.1 to 101.6 ± 3.9 µg AAE per mg) and FRAP (97.6 ± 2.3 to 127.9 ± 2.7 µg AAE per mg), with increases ranging from 192% to 232% and 1507% to 1612%, respectively. Ce–Zn Nc showed notable antioxidant capacity, followed by ZnNPs and CeNPs, all significantly elevated *versus* control. Overall, RS extract and Ce–Zn Nc exhibited superior phytochemical content and antioxidant potential, highlighting their promise as potent natural antioxidants.

### 3.9. Antioxidant radical scavenging activities

#### 3.9.1. DPPH, ABTS, and H_2_O_2_ radical scavenging activities

**Fig. 8:**
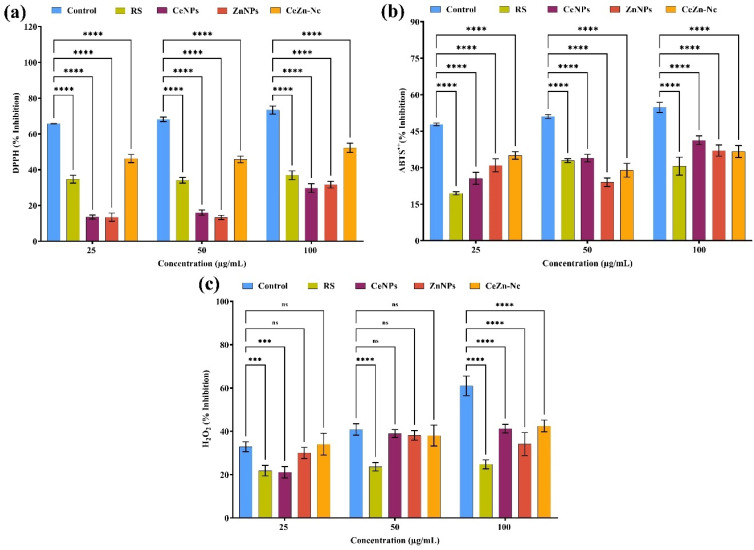
Radical scavenging activities of RS, CeNPs, ZnNPs, and Ce–Zn Nc at different concentrations (25, 50, 100 µg mL^−1^): (a) DPPH radical scavenging activity (% inhibition), (b) ABTS radical scavenging activity (% inhibition), and (c) H_2_O_2_ scavenging activity (% inhibition). Data are presented as mean ± SD (*n* = 3). Statistical significance was determined using two-way ANOVA followed by Dunnett's multiple comparison test (***p < 0.001, ****p < 0.0001, ns = not significant).

DPPH radical scavenging activity (% inhibition) was significantly lower in all samples compared to control at 25, 50, and 100 µg mL^−1^ concentrations (****p < 0.0001, Fig. 8a). Control samples showed the highest inhibition with values of 65.8 ± 0.4%, 68.2 ± 1.2%, and 73.4 ± 2.3%, respectively. Among treatments, the CeZn-Nc exhibited the greatest scavenging activity (46.3 ± 2.3% to 52.3 ± 2.6%), followed by RS with moderate inhibition (34 ± 1.6% to 37 ± 2.4%). ZnNPs and CeNPs showed significantly lower activity (13.5 ± 1.1% to 31.7 ± 1.9%). Similarly, ABTS radical scavenging activity (Fig. 8b) was significantly reduced in all samples *versus* control at all concentrations (****p < 0.0001). Control values ranged from 47.8 ± 0.6% to 54.8 ± 2.1%. Ce–Zn Nc demonstrated the highest scavenging (35.1 ± 1.6% to 36.7 ± 2.5%), followed by ZnNPs (24 ± 1.7% to 31.7 ± 1.9%). CeNPs and RS showed comparatively lower inhibition. H_2_O_2_ scavenging activity (Fig. 8c) varied significantly among samples. Controls had the highest inhibition (32.90 ± 2.28% to 61.02 ± 4.53%). Ce–Zn Nc showed notable activity (34.1 ± 5.1% to 42.5 ± 2.7%), outperforming RS (21.9 ± 2.4% to 24.8 ± 2.1%), while ZnNPs and CeNPs exhibited moderate inhibition. Significant differences compared to control were observed at 100 µg mL^−1^ (****p < 0.0001), with some lower concentration comparisons not significant (ns). Collectively, these results demonstrate enhanced radical scavenging potential of Ce–Zn Nc relative to other treatments, particularly at higher concentrations.

### 3.10. Biocompatibility and enzyme inhibition assays

#### 3.10.1. Assessment of hemolytic, anti-arthritic, cytotoxic, and alpha-amylase inhibitory activities

**Fig. 9:**
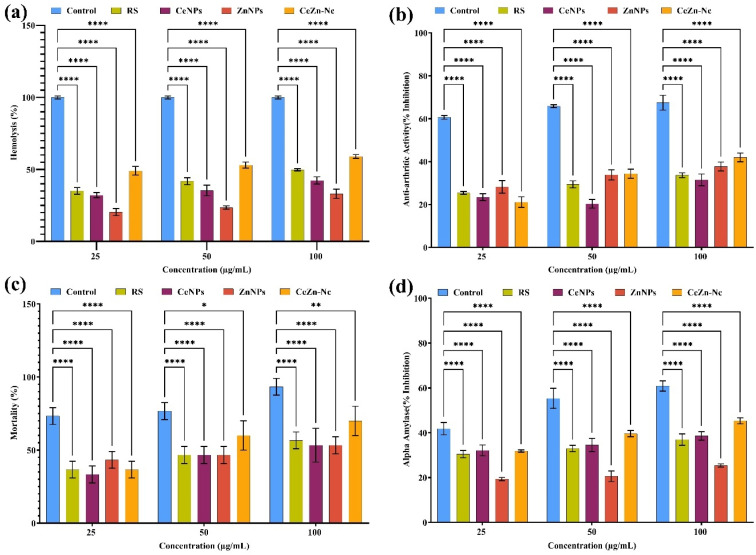
Evaluation of bioactivity and toxicity of RS, CeNPs, ZnNPs, and Ce–Zn Nc at concentrations of 25, 50, and 100 µg mL^−1^: (a) hemolytic activity (%), (b) anti-arthritic activity (% inhibition), (c) brine shrimp lethality (% mortality), and (d) alpha-amylase inhibitory activity (% inhibition). Data are expressed as mean ± SD (*n* = 3). Statistical significance determined by two-way ANOVA with Dunnett's multiple comparison test (*p < 0.05, **p < 0.01, ***p < 0.001, ****p < 0.0001).

The hemolytic activity of the synthesized materials was evaluated to assess their impact on red blood cell membrane integrity. As shown in Fig. 9a, all treatment groups exhibited significantly lower hemolysis compared to the Positive Control (100% lysis). However, the CeZn-Nc demonstrated substantial hemolytic activity, ranging from 49.1 ± 3% to 59.1 ± 1.3% as the concentration increased from 25 to 100 µg mL^−1^. In comparison, the individual ZnNPs showed the lowest hemolytic effect (20.4 ± 2.5% to 33.1 ± 3.2%). These results indicate that while the nanocomposite is less destructive than the positive control, it exceeds the standard 5% biocompatibility threshold. This suggests that the current formulation of CeZn-Nc possesses significant membrane-disrupting properties, which may be advantageous for its antibacterial and anticancer potential but necessitates further surface functionalization or dosage optimization to enhance its hemocompatibility for systemic applications.Anti-arthritic activity (% inhibition) also significantly decreased across all treatments compared to control (****p < 0.0001, Fig. 9b). Control samples recorded the highest inhibition (60.7 ± 0.9% to 67.5 ± 3.5%), while ZnNPs and Ce–Zn Nc demonstrated moderate activity (21.2 ± 2.5% to 42 ± 2%). RS and CeNPs exhibited comparatively lower inhibition (20.4 ± 2% to 33.7 ± 1.1%). Brine shrimp lethality assay revealed significantly lower mortality (%) in all treated groups *versus* the control (Fig. 9c). Control mortality was highest (73.3 ± 5.8% to 93.3 ± 5.8%), whereas Ce–Zn Nc showed moderate mortality (36.7 ± 5.8% to 70 ± 1%), significantly less than control (****p < 0.0001 at 25 and 50 µg mL; **p < 0.01 at 100 µg mL^−1^). RS, CeNPs, and ZnNPs exhibited substantially lower mortalities (20.4 ± 2.1% to 56.7 ± 5.8%). Alpha-amylase inhibitory activity (% inhibition) followed a similar trend (****p < 0.0001, Fig. 9d). Controls showed the highest inhibition (41.9 ± 2.7% to 60.9 ± 2.3%), with Ce–Zn Nc displaying the greatest inhibition among treatments (31.9 ± 0.5% to 45.3 ± 1.4%). CeNPs and RS had moderate inhibitory effects, and ZnNPs showed the lowest activity. These results indicate that CeZn-Nc exhibits significant dose-dependent hemolytic and anti-arthritic activity. While more potent than individual nanoparticles, all samples remained significantly less destructive than the positive controls.

### 3.11. Catalytic degradation and kinetic analysis

**Fig. 10:**
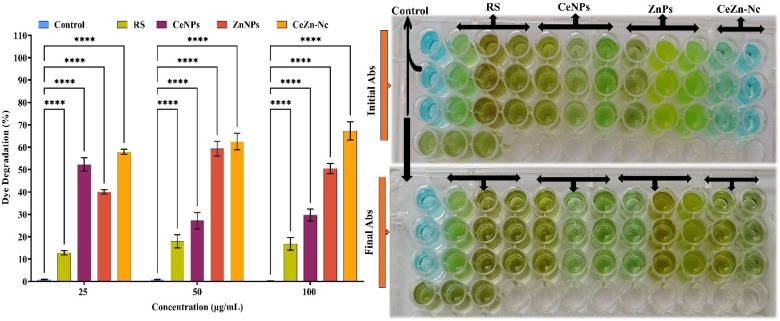
Catalytic dye degradation activity of RS, CeNPs, ZnNPs, and Ce–Zn Nc at concentrations of 25, 50, and 100 µg mL^−1^: percentage of dye degradation, and visual representation of dye discoloration before (initial absorbance) and after (final absorbance) treatment. Data are presented as mean ± SD (*n* = 3). Statistical significance was determined by two-way ANOVA with Dunnett's multiple comparisons test (****p < 0.0001 *vs.* control).

The catalytic degradation of Methylene Blue (MB) dye by RS extract, CeNPs, ZnNPs, and Ce–Zn Nc was evaluated at concentrations of 25, 50, and 100 µg mL^−1^ (Fig. 10). Control samples exhibited negligible dye degradation (<1%), confirming the stability of the dye without treatment. Quantitative analysis revealed that Ce–Zn Nc demonstrated the highest catalytic efficiency, with degradation percentages increasing from 57.9 ± 1.2% to 67.3 ± 4% across the tested concentrations (****p < 0.0001). ZnNPs showed moderate activity, ranging from 40.1 ± 1% to 50.5 ± 2.3%, while CeNPs ranged from 27.2 ± 2.9% to 52.3 ± 2.9%. The RS extract exhibited the lowest potential, with values between 12.9 ± 1% and 17.9 ± 2.9%. These results were visually corroborated by the pronounced discoloration of the dye solutions, particularly in the Ce–Zn Nc treated groups. To further understand the reaction dynamics, a pseudo-first-order kinetic model was applied to the experimental data using the equation ln(*C*_0_/*C*_*t*_) = *kt*. The calculated kinetic parameters, including the fraction remaining (*C*_0_/*C*_*t*_), the logarithmic concentration ratio ln(*C*_0_/*C*_*t*_), and the rate constant (*k*), are summarized in Table 2. The Ce–Zn Nc exhibited superior kinetic performance, achieving a maximum rate constant of 0.0093 min^−1^ at 100 µg mL^−1^ with a high correlation coefficient (*R*^2^ = 0.98). This rate is significantly higher than those of ZnNPs (0.0058 min^−1^) and CeNPs (0.0029 min^−1^) at the same concentration, confirming the synergistic enhancement of the bimetallic heterojunction (Fig. 10). The industrial and environmental viability of the Ce–Zn Nc was assessed through a three-cycle reusability test at 100 µg mL^−1^. The nanocomposite maintained remarkable stability, with degradation efficiency only slightly decreasing from 67.3% in the first cycle to 60.9% in the third cycle. This high retention of catalytic activity (over 90%) highlights the structural integrity of the green-synthesized nanomaterial and the effective stabilization provided by the *R. stricta* phytochemicals. Statistical analysis confirmed significant differences between all nanoparticle treatments and the control (****p < 0.0001), underscoring the potential of Ce–Zn Nc for high-performance dye remediation.

**Table 2:** Kinetic parameters for the pseudo-first-order degradation of Methylene Blue (MB) using *R. stricta* extract, CeNPs, ZnNPs, and Ce–Zn Nc at various concentrations

Treatment	Concentration (µg mL^−1^)	*C*_*t*_/*C*_0_	ln(*C*_*t*_/*C*_0_)	Degradation (%)	Rate constant (*k* min^−1^)	Correlation (*R*^2^)
RS extract	25	0.8715	0.1375	12.65%	0.0011	0.95
	50	0.8203	0.1918	17.97%	0.0016	0.96
	100	0.8315	0.1845	16.85%	0.0015	0.94
CeNPs	25	0.4769	0.7405	52.31%	0.0062	0.97
	50	0.7284	0.3169	27.16%	0.0026	0.96
	100	0.7031	0.3522	29.69%	0.0029	0.98
ZnNPs	25	0.5988	0.5128	40.12%	0.0043	0.98
	50	0.4070	0.8989	59.30%	0.0075	0.99
	100	0.4948	0.7036	50.52%	0.0058	0.97
Ce–Zn Nc	25	0.4208	0.8656	57.92%	0.0072	0.98
	50	0.3748	0.9813	62.52%	0.0082	0.99
	100	0.3270	1.1177	67.30%	0.0093	0.98

### 3.12. Antibacterial activity

**Fig. 11:**
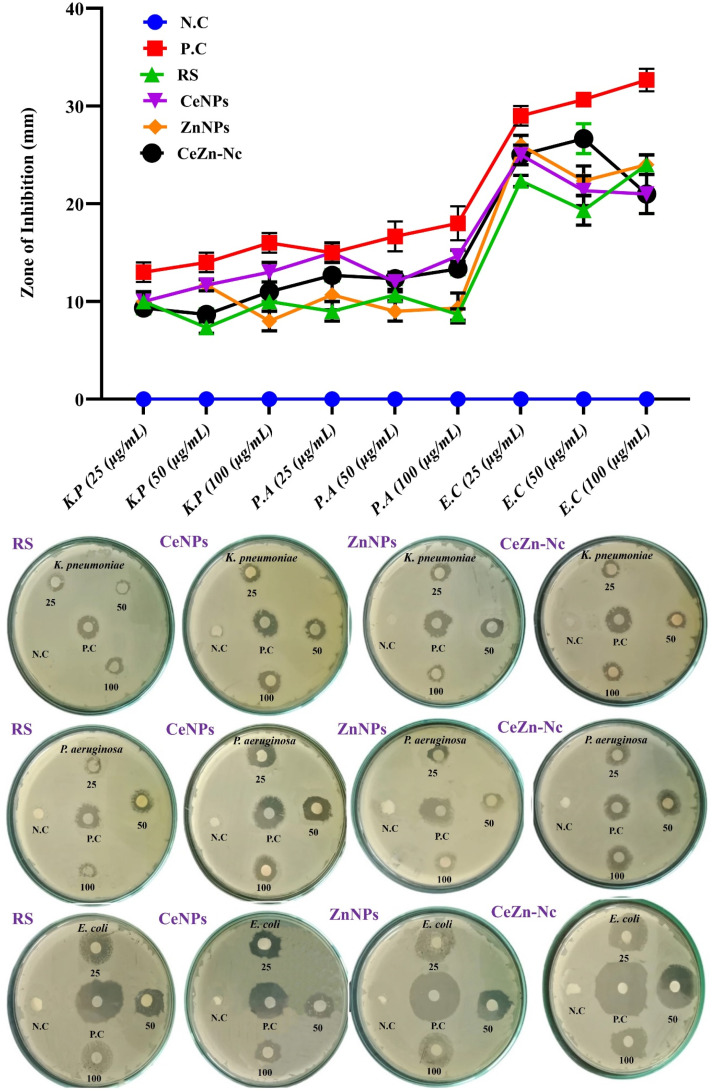
Antibacterial activity of RS, CeNPs, ZnNPs, and CeZn-Nc. The plot and corresponding agar plates illustrate the dose-dependent inhibition zones (mm) at treatment concentrations of 25, 50, and 100 µg mL^−1^. Error bars in the graph represent the standard deviation (SD) for *n* = 3, with P.C (Positive Control: Ampicillin) and N.C (Negative Control: TSB) used as benchmarks for comparison.

The antibacterial efficacy of *R. stricta* (RS) extract, CeNPs, ZnNPs, and CeZn-Nc was evaluated against *K. pneumoniae*, *P. aeruginosa*, and *E. coli* at concentrations of 25, 50, and 100 µg mL^−1^. As illustrated in Fig. 11, the Negative Control (N.C), consisting of Tryptic Soy Broth (TSB), exhibited no zone of inhibition (0 mm) across all tested bacterial strains, confirming that the growth medium does not possess inherent antimicrobial activity. The Positive Control (P.C), represented by the standard antibiotic Ampicillin, showed the highest inhibitory zones, ranging from 13 ± 1 mm to 32.7 ± 1.2 mm. Among the experimental groups, the CeZn-Nc and ZnNPs demonstrated robust antibacterial activity, particularly against *E. coli*, where the CeZn-Nc reached a significant inhibition zone of 26.7 ± 1.5 mm at 50 µg mL^−1^. These results were found to be highly significant compared to the negative control across all concentrations, emphasizing the potent, dose-dependent antibacterial nature of the synthesized nanocomposite.

## 4. Discussion

The GC-MS profiling of *R. stricta* leaf extract in this study revealed a diverse range of bioactive phytochemicals, including fatty acids, phenolics, alkaloids, and terpenoids, which is in line with previous findings by,^31^ who emphasized the plant's rich phytochemical profile and therapeutic potential and these compounds act as natural reducing and stabilizing agents. The novelty of our approach lies in the identification of specific molecular drivers, such as Quebrachamine and Palmitic acid, which act as site-specific natural reducing and stabilizing agents. Supporting this,^29^ identified similar phytochemical classes, such as phenolic acids, alkaloids, and fatty acid esters, in *R. stricta* ethanolic extracts, linking them to the successful biosynthesis of silver nanoparticles with notable antimicrobial activity. This aligns with our findings, where the phytochemical-rich *R. stricta* extract facilitated the green synthesis of CeNPs, ZnNPs, and Ce–Zn nanocomposites, as confirmed by UV-Vis spectroscopy through distinct SPR peaks. The observed red shift in the Ce–Zn SPR peak suggests electronic interactions between cerium and zinc oxides, indicative of nanocomposite formation. The XRD findings are in close agreement with the results reported by,^44^ who identified the cubic fluorite phase of cerium oxide using JCPDS 43-1002 and noted that peak positions remained stable across varying calcination temperatures. Furthermore, the reduction in crystallite size and the successful formation of a bimetallic interface align with the observations of,^34^ where the green synthesis of CeO_2_@ZnO nanocomposites resulted in high phase purity and enhanced surface properties. This structural synergy between the two oxides is a critical factor in achieving the superior catalytic and antioxidant efficiencies observed in this study, mirroring the multifunctional performance reported in recent literature.

Further validation was provided by FTIR and Raman spectroscopy, which identified key functional groups, such as hydroxyl, carbonyl, and amines, participating in nanoparticle formation and stabilization. SEM and HR-TEM analyses confirmed the successful synthesis of nanoparticles with distinct morphologies: aggregated spherical CeNPs, porous flaky ZnNPs, and compact, uniformly distributed Ce–Zn nanocomposites. The reduced average particle size of the Ce–Zn Nc (∼10.7 nm) reflects improved morphological control and enhanced homogeneity, consistent with findings by.^45^ The lattice fringes observed in TEM images confirm the crystalline nature of the synthesized nanomaterials, which supports their potential in catalytic and biomedical applications.^46^

A critical factor in the enhanced performance of the Ce–Zn Nc is the synergistic electronic environment created at the bimetallic interface. The incorporation of Ce into the ZnO lattice facilitates a dynamic Ce^3+^/Ce^4+^ redox cycle.^47^ According to the Kröger–Vink notation, the reduction of Ce^4+^ to Ce^3+^ induces the formation of oxygen vacancies (V_O_) to maintain charge neutrality.^48,49^ These vacancies serve as potent “active sites” for the adsorption of dye molecules and oxygen species. Furthermore, the observed red-shift in our UV-Vis data confirms the formation of a Type-II heterojunction. In this configuration, the staggered band alignment allows electrons to migrate from the conduction band of ZnO to CeO_2_, while holes accumulate in the valence band of ZnO. This spatial separation effectively suppresses electron–hole recombination, ensuring a higher flux of charge carriers for surface redox reactions.^50,51^ These morphological and structural observations strongly agree with reports by ref. 52 and 34, who described well-defined crystalline CeO_2_–ZnO nanocomposites with agglomerated but uniform morphologies. FTIR spectral features in our study, including Zn–O and Ce–O stretching vibrations, also mirror those reported in these studies, affirming successful composite formation. Additionally, the red-shifted absorption and narrowed band gap in the Ce–Zn nanocomposites align with,^34^ indicating enhanced optical properties due to Ce^4+^ incorporation and improved charge separation. TGA-DSC analysis further revealed the superior thermal stability of the Ce–Zn nanocomposite compared to individual nanoparticles, likely due to synergistic interactions between the metal oxides, as similarly noted in bimetallic systems by.^46^

The superior total flavonoid and phenolic contents in the RS extract and Ce–Zn Nc corresponded with significantly elevated antioxidant capacities, as evidenced by TAC and FRAP assays. This corroborates prior work showing that plant extracts rich in phenolics and flavonoids contribute substantially to antioxidant potential, and that nanocomposites can enhance this effect by improving surface interactions and stability.^53^ Notably, the higher antioxidant activity of Ce–Zn Nc relative to single metal oxide NPs stems from the aforementioned Ce^3+^/Ce^4+^ redox cycling, which enhances electron donation and free radical scavenging abilities. Our findings are also supported by ref. 54, who reported that CeO_2_ NPs synthesized using *Tribulus terrestris* exhibited dose-dependent DPPH radical scavenging activity, reaching up to 88.2%, and by,^55^ who linked high antioxidant performance of Au–ZnO nanocomposites to the presence of phytochemical-rich *Artemisia* extracts. DPPH, ABTS, and H_2_O_2_ scavenging assays in our study consistently showed that Ce–Zn Nc had enhanced radical scavenging activity compared to CeNPs and ZnNPs, with RS extract showing moderate effects. These results align with existing studies where cerium and zinc-based nanomaterials demonstrated potent antioxidant activities by neutralizing reactive oxygen species (ROS), thereby protecting against oxidative stress.^56^

The Ce–Zn Nc demonstrated favorable biocompatibility, as evidenced by low hemolytic activity and reduced brine shrimp cytotoxicity.^57,58^ The nanocomposite also showed strong anti-arthritic activity and the highest α-amylase inhibition, surpassing CeNPs and ZnNPs, and aligning with findings from ref. 59 and 60. The enhanced enzyme inhibitory activity is likely due to the synergistic interaction of Zn^2+^/Ce^4+^ ions with the thiol and amino groups of the enzymes, a process stabilized by the *R. stricta* metabolites. The Ce–Zn Nc demonstrated superior catalytic degradation of methylene blue dye (67.3%) compared to individual nanoparticles. This is mechanistically driven by the accelerated generation of ROS, specifically hydroxyl radicals (OH) and superoxide anions (O_2_^−^). The trapped electrons on the CeO_2_ surface reduce dissolved oxygen to O_2_^−^, while holes in the ZnO phase oxidize H_2_O to OH. These radicals non-selectively attack the chromophore bonds of the dye.^61,62^ The Ce–Zn Nc demonstrated superior catalytic degradation of methylene blue dye compared to individual metal oxide nanoparticles and RS extract, likely due to enhanced surface area, reduced electron–hole recombination, and improved electron transfer efficiency. These findings are in agreement with,^63^ who reported 98.1% degradation of Eosin Y using Ce-doped ZnO under optimized conditions, and,^64^ who achieved complete methyl green degradation with CeO_2_–ZnO composites within 30 minutes. The enhanced photocatalytic activity in our Ce–Zn Nc also mirrors the work of,^65^ where co-doping with Ce significantly boosted degradation efficiencies for dyes like Congo red and Rhodamine B. These studies collectively highlight the crucial role of cerium incorporation in boosting the photocatalytic potential of ZnO-based nanomaterials, supporting our findings and their relevance for environmental remediation applications. As summarized in Table 3, the photocatalytic efficiency and kinetic rate constant (*k* = 0.0093 min^−1^) of the synthesized CeZn-Nc are highly competitive with other recently reported green-synthesized metal oxide systems. Notably, our nanocomposite achieved a significantly smaller particle size (10.7 nm) compared to *Withania coagulans* and *Pavonia zeylanica* mediated catalysts, highlighting the superior capping and stabilizing efficiency of the *Rhazya stricta* extract.

**Table 3:** Comparison of photocatalytic performance and kinetic rate constants of Ce–Zn-Nc with other green-synthesized metal oxide nanomaterials for MB degradation

Catalyst system	Plant extract source	Particle size (nm)	Time (min)	Efficiency (%)	Rate constant (*k*, min−1)	References
CeZn-Nc	*Rhazya stricta*	10.7	120	67.30%	0.0093	This work
ZnO NPs	*Pavonia zeylanica*	19.58	120	89.32%	0.016	71
Co_3_O_4_–ZnO NCs	*Calpurnia aurea*	∼25–35	60	98.06%	0.061	72
CeO_2_@ZnO	*Azadirachta indica*	9.0–27.0	120	94%	0.015	34
ZnO/CeO_2_ (ZC-4)	*Hydrothermal*	∼20–30	60	56.86%	0.0020	73
CeO_2_–ZnO	*Acacia nilotica*	∼20–30	170	80%	0.0094	62
Ce–ZnO (5%)	*Withania coagulans*	18.87	100	85%	0.0190	74

Finally, the Ce–Zn Nc exhibited superior antibacterial activity against *K. pneumoniae*, *P. aeruginosa*, and *E. coli*. This effect is attributed to a “synergistic oxidative burst” where the bimetallic interface promotes higher levels of lipid peroxidation and membrane disruption than individual oxides.^66^ The identified metabolites like Quebrachamine potentially enhance the permeability of bacterial membranes, allowing the localized ROS and metal ions to cause irreversible damage to internal cell structures. These results confirm the strong potential of Ce–Zn Nc for combating multidrug-resistant pathogens^67,68^.^66^ reported strong inhibition zones, especially for ZnO–CeO_2_ (2 : 8) nanocomposites against *Staphylococcus aureus*, while^69^ observed up to 35 mm inhibition against *E. coli* with green-synthesized Ce–Zn composites. The enhanced antibacterial effect is attributed to synergistic interactions between Ce and Zn, promoting ROS generation, metal ion release, and membrane disruption. Similar conclusions were drawn by,^70^ who noted significant bacterial inhibition by green ZnO NPs due to phytochemical-mediated bioactivity. These results confirm the strong potential of Ce–Zn Nc for combating multidrug-resistant pathogens. The green-synthesized Ce–Zn Nc demonstrated multifunctional properties, including strong antioxidant, antimicrobial, catalytic, and enzyme inhibitory activities, and acceptable biocompatibility. These findings highlight their broad potential for biomedical and environmental applications, supporting the sustainable development of plant-based nanomaterials.

## 5. Conclusions

This study successfully demonstrated a sustainable, plant-mediated synthesis of CeO_2_, ZnO, and their synergistic Ce–Zn nanocomposite using *R. stricta* leaf extract, effectively eliminating the need for hazardous chemical reductants. Comprehensive phytochemical profiling *via* GC-MS identified the bioactive role of natural stabilizers, supporting a circular-economy approach to nanomaterial production. The resulting crystalline materials showed high thermal stability and multifunctional efficacy, with the Ce–Zn nanocomposite exhibiting superior synergistic performance in antioxidant, antibacterial, and catalytic applications compared to its individual components. Specifically, its significant degradation of organic dyes and low hemolytic toxicity provide a quantifiable, eco-friendly solution to challenges outlined in SDG 3 (Good Health), SDG 6 (Clean Water), and SDG 12 (Responsible Production). Ultimately, this work highlights the potential of bio-inspired nanotechnology to replace energy-intensive synthetic routes with stable, biocompatible, and high-performance materials for environmental remediation and biomedical therapy.

## Ethics approval and consent to participate

This study did not involve research on human subjects, animals, or endangered species. The collection of *Rhazya stricta* was performed on private land in Village Sawans, District Mianwali, Punjab Province, Pakistan, at coordinates 32°43′44″ N, 71°37′59″ E, with an elevation of 285 meters above sea level, and with explicit permission from the landowner. The research and field studies on *R. stricta*, including the collection of plant material, fully comply with the relevant institutional, national, and international regulations. We have ensured that all procedures were in line with the applicable ethical guidelines for plant collection. Additionally, we have carefully followed the IUCN Policy Statement on Research Involving Species at Risk of Extinction and the Convention on the Trade in Endangered Species of Wild Fauna and Flora (CITES). Since *R. stricta* is not listed as a species at risk of extinction, these guidelines were not specifically applicable. However, we have adhered to all general ethical practices for plant collection and research.

## Author contributions

Amjid Khan: conceptualization, visualization, data curation, investigation, methodology, software, funding acquisition, data validation, and writing – original draft. Tauqeer Ahmed Qadri: data curation, formal analysis, software, and writing – review & editing. Dilawar Hassan: data curation, formal analysis, resources, validation, and writing – review & editing. Rashid Abbas Khan and Bushra Ashiq: formal analysis, data curation, resources, and writing – review & editing. Ayesha Sani: data curation, formal analysis, resources, validation, and writing – review & editing. Malik Maaza and Zabta Khan Shinwari: project administration, funding acquisition, supervision, validation, and writing – review & editing. All authors read and approved the final manuscript.

## Conflicts of interest

The authors declare that they have no known competing financial interests or personal relationships that could have appeared to influence the work reported in this paper.

## Supplementary Material

RA-016-D6RA02267G-s001

## Data Availability

The data supporting this article are provided in supplementary information (SI). Supplementary information: characterization data, specifically FTIR absorption band assignments (Table S1) and Raman shift vibrations (Table S2) for the RS extract and synthesized nanoparticles. See DOI: https://doi.org/10.1039/d6ra02267g.
