# Supplementary material for: Sustainable green synthesis of CeO2–ZnO nanocomposites using Rhazya stricta: enhanced synergistic solutions for biomedical challenges and environmental remediation
Source: RSC Adv. 2026 May 6;16(26):23370–90. doi: 10.1039/d6ra02267g (PMC13147360; doi:10.1039/d6ra02267g)
Supplement: RA-016-D6RA02267G-s001 [file RA-016-D6RA02267G-s001.pdf]

Supplementary Information (SI) for RSC Advances.
This journal is © The Royal Society of Chemistry 2026

Sustainable Green Synthesis of CeO_2-ZnO Nanocomposites using *Rhazya stricta*: Enhanced Synergistic Solutions for Biomedical Challenges and Environmental Remediation

Amjid Khan[1,2,3]*, Tauqeer Ahmed Qadri[4], Rashid Abbas Khan[1,2,3], Dilawar Hassan[1,2,3], Bushra Ashiq[5], Ayesha Sani[1,2,3], Zabta Khan Shinwari[6], Malik Maaza[1,2,3]*

[1]UNESCO-UNISA Africa Chair in Nanosciences and Nanotechnologies, College of Graduate Studies, University of South Africa, 1 Preller Street, Muckleneuk Ridge, P.O. Box 392, Pretoria, Gauteng Province, 0003, South Africa

[2]Nanosciences African Network (NANOAFNET), Materials Research Department, iThemba LABS-National Research Foundation, Western Cape Province, South Africa

[3]African Centre of Competencies in Enhanced Nanosciences & Nanotechnologies for SDGs (ACCENTS), 1 Preller Street, Muckleuneuck Ridge, P.O. Box 392, Pretoria, Gauteng Province, 0003, South Africa

[4]Department of Biosciences, COMSATS University Islamabad, Islamabad-45550, Pakistan

[5]Department of Biomedical Engineering, Research Center for Nano-biomaterials & AMP; Regenerative Medicine, College of Artificial Intelligence, Taiyuan University of Technology, Taiyuan 030024, PR China

[6]Department of Plant Sciences, Faculty of Biological Sciences, Quaid-i-Azam University, Islamabad, 45320, Pakistan

*** Correspondence:**
Amjid Khan. Email: khana2@unisa.ac.za
Malik Maaza. Email: maazam@unisa.ac.za

Table S1. FTIR Absorption Bands and Functional Group Assignments of RS Extract, CeNPs, ZnNPs, and Ce-Zn Nc.

Sample	Wavenumber (cm^{-1})	Assigned Functional Group (s)	Possible Chemical Bonds / Vibrations
**RS**	2925	C–H stretching	Alkanes/Terpenoids
	1645	C=O / N–H Stretching	Amide I (Proteins)
	1052	C–O stretching	Ethers/Alcohols
**CeNPs**	3392	O–H stretching	Capping interaction
	2913	C–H stretching	Alkanes
	1612	C=O stretching	Carbonyl groups
	523	Ce–O Stretching	Cerium Oxide formation
**ZnNPs**	3371	O–H stretching	Polyphenol involvement
	1602	C=O stretching	Amides/Carbonyls
	544	Zn–O stretching	Zinc Oxide formation
**Ce-Zn Nc**	3292	O–H Stretching	Capping stabilization
	1705	C=O Stretching	Carboxylic acids
	1516	Aromatic C=C Stretching	Flavonoids/Aromatic rings
	515	M–O Stretching	Bimetallic Oxide junction

Table S2. Raman Shift Bands and Functional Group Assignments of CeNPs, ZnNPs, and Ce-Zn Nc.

Sample	Raman Shift (cm^{-1})	Assigned Vibrations	Possible Chemical Bonds / Functional Groups
**CeNPs**	70	Lattice vibrations	Metal oxide lattice modes
	2919	C–H stretching	Alkanes/Methyl groups
	3525	O–H / N–H stretching	Hydroxyl/Amine groups
**ZnNPs**	76	Lattice vibrations	Metal oxide lattice modes
	3017	C–H stretching	Alkanes/Capping residues
	3526	O–H stretching	Hydroxyl groups
**Ce-Zn Nc**	68	Lattice vibrations	Bimetallic lattice modes
	2927	C–H stretching	Alkanes
	3411	O–H / N–H stretching	Hydroxyl/Amine groups
